# Does provision of targeted health care for the unemployed enhance re-employment?

**DOI:** 10.1186/1471-2458-14-1200

**Published:** 2014-11-21

**Authors:** Katri Romppainen, Antti Saloniemi, Ulla Kinnunen, Virpi Liukkonen, Pekka Virtanen

**Affiliations:** P.O Box 607, 33014 Tampere, Finland; Department of Pori, School of Social Sciences and Humanities, University of Tampere, Pori, Finland; School of Social Sciences and Humanities, University of Tampere, Tampere, Finland; School of Health Sciences, University of Tampere, Tampere, Finland

**Keywords:** Health services, Access to health care, Unemployment

## Abstract

**Background:**

There is increasing pressure to develop services to enhance the health of the workforce on the periphery of the labour market. Health promotion among unemployed people may improve their health but also to increase their employability. We tested whether re-employment can be enhanced with a health care intervention targeted at the unemployed.

**Methods:**

A 3-year follow-up, controlled design was used. The data were collected among unemployed people (n = 539) participating in active labour market policy measures. The baseline survey included established habitually used health questionnaires. The intervention consisted of three health check-ups and on-demand health services. Logistic regression analyses were used to obtain the odds ratios of the intervention group versus control group for being re-employed at follow-up. Health-related differences in the re-employment effects of the intervention were assessed through the significance of the interaction in the regression analyses.

**Results:**

The intervention did not serve to improve re-employment: at follow-up 50% of both the intervention group and the control group were at work. In further analyses, the odds ratios showed that the intervention tended to improve re-employment among participants in good health, whereas an opposite tendency was seen among those with poor health. The differences, however, were statistically non-significant.

**Conclusion:**

The experimental health service did not show any beneficial effects on re-employment. Nevertheless, rather than considering any particular health care as unnecessary and ineffective, we would like to stress the complexity of providing health services to match the diversity of the unemployed.

## Background

Unemployment is associated with poor mental and physical health independent of time and place [1, 2]. It is well understood that the antithesis ‘selection or causation’ is false [3]. Both selective and causal processes are therefore relevant in considering the preventive and the illness-related health services for the working aged population. Policymakers and health service researchers also consider health care for the unemployed a major challenge, but there are concerns about appropriate arrangements and the consequent effectiveness and efficiency of the service.

Implementing a service always takes place in national structures and is actualised in different contexts. The present study reporting a health care intervention among the unemployed was conducted in Finland, where unemployment entails a specific risk of being excluded from health care [4]. The reason for this is the structure of the primary health care service for working aged people, which largely relies on occupational health care services (OHC) [5]. OHC is provided by law for all waged and salaried employees, and has developed beyond worker protection and occupational medicine into a comprehensive system of illness-related and preventive general health care [6]. About 90% of the employed population has access to this service [7] which, however, is lost together with the job. In the other words, as a consequence of unemployment the citizen also loses part of his/her health service system and the available service is limited mainly to communal Health Centres. Therefore there is a particular pressure to develop services to cater for the workforce on the periphery of the labour market. Extensive development projects have been piloted in order to find ways to implement health services for the unemployed [8], and under the amendment to the Health Care Act of 2011 municipalities are obliged to provide health promotion and check-ups for the working aged who do not have access to OHC. Research evidence of the impacts of such a service is still lacking. Also internationally, publications tend to write about the state of affairs and recommend novel services [9–12], whereas studies concentrating on the provision and effects of the services are scarce [e.g. [13–15].

Although the ultimate and explicit aim of health care interventions is to prevent prolongation of the unemployment, to the best of our knowledge there are no studies with re-employment as the outcome of the service. The opposite phenomenon, i.e. the ‘indirect effects’ [16] of employment policy interventions on health, has been studied more extensively. The evidence that the interventions are able to promote health is limited, and they seem to be relatively ineffective even with respect to work participation [17]. With these research defects as the starting point, we ask in the present study if the re-employment effect of active labour market measures could be improved by accompanying health services.

The idea was to apply the existing Finnish OHC as a health service for unemployed people participating in active labour market policy (ALMP) measures. We wanted to carry over the principles and professionals of OHC to serve the unemployed in an attempt to enhance their employability. The OHC-ALMP setting was chosen in order to reduce the transfer problem of an evidence-based intervention from research settings to real contexts and populations. The aim of our trial was to investigate whether a health intervention among unemployed people enhances their re-employment prospects compared to those left without the service. In particular, we were interested in studying whether the possible effect depends on the self-perceived health of the unemployed individuals.

## Methods

Career Health Care (CHC) was an intervention resembling Finnish OHC services, except that the clients were recruited from jobseekers participating in ALMP measures (vocational training courses, subsidized employment, and participatory training for entering the labour market). Adopting the logics of OHC, CHC aimed to tackle the problems and risks inherent in unemployment. Six occupational health nurses from established OHC providers in three localities in southern and central Finland were recruited for the client work. The CHC service consisted of three health check-ups by the nurses at the beginning and end of the ALMP measure, and when three years had elapsed since the beginning. Main focus of CHC was on health promotion and primary prevention, managed with a specific ‘health plan’ adopted from OHC [6]. The activities consisted health screenings, assessment of client’s working ability and individual health promotion-oriented guidance and counselling, emphasising in particular the health-related risks and problems during the unemployment spell. The instructions for organising the check-ups were, however, left relatively open, and nurses were encouraged to vary and develop the encounters (see [18] for more details).

In addition to personal interviews, the nurses received information through the questionnaires that the clients returned at the check-up. Moreover, the encounter included three kinds of physical performance tests and measurement of weight, blood pressure and pulse. Regular laboratory screenings or physician consultations were not routinely included in CHC, but the needs were assessed individually and the clients received referrals and guidance to appropriate health services. The nurses could also book control visits in CHC, for instance for checking the blood pressure. The most common topics of health promotion and health counselling were ‘classic’, such as smoking cessation, excess alcohol consumption, diet due to high cholesterol, diabetes or obesity, physical exercise and psychosocial conditions. In all, the idea of CHC was to provide the clients an opportunity to use health care in maintaining their workability of and boosting their re-employment; therefore no co-work systems with social or employment services, for example, were established but the clients were encouraged to use them if needs were detected.

**Figure 1:**
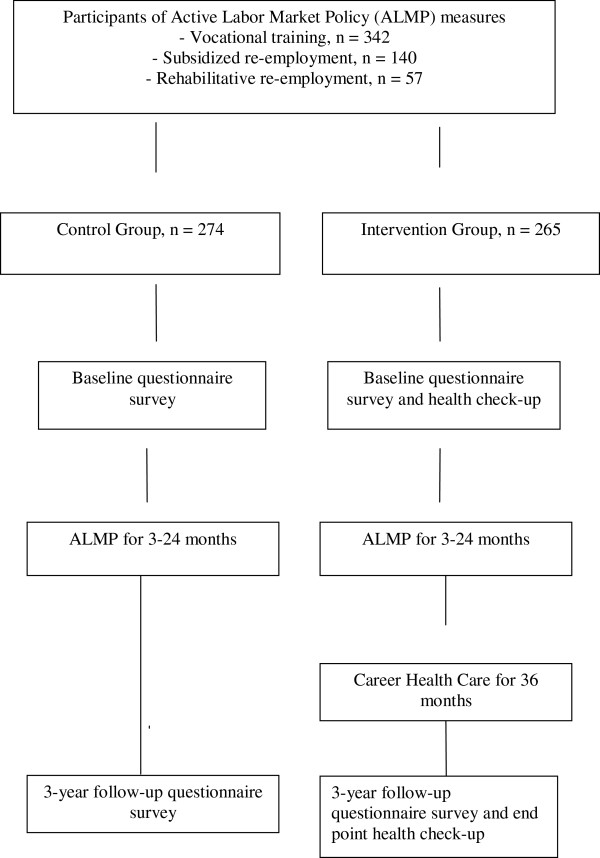
**Design of the career health care trial.**

In addition to the scheduled encounters, the participants had throughout the three-year CHC-clientship an opportunity to spontaneous illness related contacts with the nurse and, if needed, with a physician. During the encounters, the participants also discussed their background health status in light of their employment histories and vocational goals.The design of the CHC trial is presented in Figure 1. The sample of this study consisted of 539 unemployed individuals who were enrolled in ALMP in 2002 and 2003. The participants were ‘healthy’ unemployed and possible disability was not used as criterion in selection. The vocational courses were chosen purposefully in order to obtain roughly equal amount of men and women, as well as to include in the study participants with a range of educational levels, to the intervention and the control group. Participants of the subsidized employment and participatory training were randomized at individual level at the recruitment occasion. The intervention group had a privilege to extra health service targeted for the unemployed (CHC), whereas the control group only could use the regular communal Health Centres. The intervention group (n = 265) with access to CHC and also the control group (n = 274) was recruited from among voluntary participants at the beginning of the ALMP measures. The researchers visited a lesson at the beginning of the vocational training courses and informed the participants about the study. Those who consented filled out a questionnaire. Participants of subsidized employment and participants in training for entering the labour market got a written information about opportunity to join the study, and those who consented were randomized. Baseline data were collected in the period 2002–2003 (Time 1) with questionnaires at the beginning of the ALMP programmes. A follow-up survey was conducted three years later (Time 2): the participants of the intervention group completed the questionnaire at the health check-up; participants of the control group returned the questionnaire by post.

The study had a steering group consisting of representatives of the Ministry of Labour and Ministry of Social Affairs and Health. At recruitment all participants received spoken and written information about the study, where it was made explicit that enrolment was voluntary and not a condition for participation in ALMP and the associated benefits. At the time of planning and implementation of the study, the Medical Research Act about Ethics Committees had not yet been enacted in Finland. There were Ethic Boards which, however, were oriented narrowly to biomedical experiments, and this kind of studies on health promotion services were not subjected to external ethical assessment. We asked the Ethics Committee of Pirkanmaa University Hospital District to assess retrospectively the study plan, and the committee stated that a study with corresponding design would be approvable (ETL-code R13024).

The baseline surveys included established questionnaires of perceived physical and mental health and well-being. The present study utilised four health indicators. Self-rated general health (SRH) was elicited with the response options 1 = good, 2 = fairly good, 3 = average, 4 = rather poor or 5 = poor and dichotomised to optimal (1–2) and suboptimal (3–5). Psychological distress was measured with the General Health Questionnaire (12-GHQ, case vs. not, cut-off value 3/4)) [19]. Depressiveness was evaluated using the Beck Depression Inventory (BDI) [20] and dichotomised to not/mildly depressed (cut-off value 4/5). The fourth indicator was Sense of Coherence (SOC), which was measured with a 13-item questionnaire [21] and dichotomised at the median. One question about current employment status (employed vs. not) was added into the 3-year follow-up questionnaire.

All statistical analyses were conducted using SPSS (version 19) for Windows. The level of statistical significance was set at 0.05. We used binary logistic regression analyses to obtain the odds ratio of the intervention group vs. control group for being re-employed at follow-up. The group × health interaction term of the regression analyses was used to assess the health-related differences in the re-employment effects of the intervention. Gender, age, level of vocational education and length of unemployment prior to entering the ALMP measure were controlled for as backgrounds factors.

## Results

Of the original sample (n = 539), 322 (60%) participated in the follow-up survey (Table 1). Drop-out was more common in the intervention than in the control group (49% vs. 31%). More men than women were lost to follow-up, otherwise participation was not related to the baseline variables. Among the participants at follow-up there were fewer women in the intervention group than in the control group (64% vs. 71%), whereas the groups did not differ in educational level, age and length of unemployment at baseline. Suboptimal self-rated health was more common (30% vs. 23%) in the intervention group. All mentioned differences were non-significant.

**Table 1:** **Descriptive statistics of the intervention group and the control group at baseline and at the end of the career health care experiment**

	Intervention group		Control group	
	Recruited at baseline (n = 265)	Participated at end (n = 134)	Recruited at baseline (n = 274)	Participated at end (n = 188)
Gender				
Men	41%	36%	35%	29%
Women	59%	64%	65%	71%
Former education				
University or college	20%	21%	18%	21%
Vocational school	42%	43%	43%	45%
ALMP course or none	38%	36%	39%	34%
Unemployment at baseline				
Less than 1 year	67%	66%	67%	69%
1 year or more	33%	34%	33%	31%
Mean age at baseline	38.0	39.6	37.6	38.5
Self-rated health at baseline				
Optimal	68%	70%	76%	77%
Suboptimal	32%	30%	24%	23%

Table 2 describes those who participated at the end by the group and by the four indicators of health. According to all indicators, suboptimal health was slightly more common in the intervention group.

**Table 2:** **Health of the participants of the intervention group and the control group in the beginning of the follow-up**

	Intervention group		Control group	
	Optimal	Suboptimal	Optimal	Suboptimal
Self-rated health	91 (70%)	39 (30%)	137 (77%)	42 (24%)
Depressiveness	96 (76%)	31 (24%)	135 (77%)	40 (23%)
Psychological distress	105 (82%)	23 (18%)	152 (85%)	26 (15%)
Sense of coherence	57 (45%)	69 (55%)	95 (54%)	81 (46%)

In the intervention group, 50% were employed at follow-up. The percentage was exactly the same in the control group. When regressed for background factors (gender, age, level of education, duration of unemployment), odds ratio for unemployment of the intervention group was 0.92 (95% confidence interval 0.57–1.49).

In order to investigate whether the re-employment effects of CHC depend on baseline health status, we analysed separately those with reportedly good (Table 3) and with poor (Table 4) health. Among the participants reportedly in good health, the intervention did not increase re-employment although the odds ratios with all four health indicators tended to show higher re-employment (Table 3). In the corresponding analysis of those with reportedly poor health (Table 4), the re-employment in the intervention group was lower than in the control group. However, the differences were statistically non-significant both among those reporting good and those reporting poor health.

**Table 3:** **Odds ratios with 95% confidence intervals for being employed at the end of the career health care experiment in cohorts with optimal self-rated overall health, optimal mental health (GHQ), optimal mood (BDI) and high sense of coherence at baseline**

	Employed	Model 1	Model 2
Optimal self-rated health			
- Control group	53%	1	1
- Intervention group	59%	1.28 (0.75-2.19)	1.38 (0.78-2.42)
Low psychological distress			
- Control group	54%	1	1
- Intervention group	51%	0.90 (0.55-1.49)	1.07 (0.63-1.83)
No depressive symptoms			
- Control group	51%	1	1
- Intervention group	55%	1.18 (0.70-1.99)	1.30 (0.74-2.28)
High sense of coherence			
- Control group	49%	1	1
- Intervention group	58%	1.47 (0.78-2.80)	1.79 (0.90-3.58)

Model 1: Unadjusted.

Model 2: Adjusted for gender, age, vocational education and length of unemployment at entry to the experiment.

**Table 4:** **Odds ratios with 95% confidence intervals for being employed at the end of the career health care experiment in cohorts with suboptimal self-rated overall health, suboptimal mental health (GHQ), suboptimal mood (BDI) and low sense of coherence at baseline**

	Employed	Model 1	Model 2
Suboptimal self-rated health			
- Control group	41%	1	1
- Intervention group	28%	0.58 (0.23-1.47)	0.56 (0.20-1.54)
High psychological distress			
- Control group	35%	1	1
- Intervention group	39%	1.21 (0.38-3.89)	0.90 (0.23-3.58)
Depressive symptoms			
- Control group	53%	1	1
- Intervention group	36%	0.50 (0.19-1.30)	0.42 (0.14-1.30)
Low sense of coherence			
- Control group	52%	1	1
- Intervention group	44%	0.72 (0.37-1.40)	0.72 (0.36-1.47)

Model 1: Unadjusted.

Model 2: Adjusted for gender, age, vocational education and length of unemployment at entry to the experiment.

Finally, we analysed the complete study population and utilised p-values for interaction from the fully adjusted regression models to assess whether those reporting poor health differed from those reporting good health with respect to the re-employment effects of the CHC. Regarding sense of coherence (OR 1.79 for higher vs. OR 0.72 for lower SOC) the difference was nearly significant (p-value 0.064), while the p-values for SRH (0.197), for psychological distress (0.874) and for depression (0.133) were non-significant.

## Discussion

Earlier studies and scholars have usually taken as the starting point the idea that it is possible to enhance employment by improving the health of the unemployed [e.g. [22, 23]. The setting of our study was in line with this general emphasis. Yet it is not guaranteed that changes in health lead to changes in employment as well. Therefore the direct outcome of our intervention was re-employment, i.e. at this time health care was treated as an instrument which, by definition, promotes health, in particular among those with poor health. On the other hand, we are aware of the fact that, at least in the worksite interventions, the health impacts have turned out at best very modest, and the results are mixed [24, 25].

We aimed to investigate whether re-employment can be enhanced with a health care intervention among the unemployed. No such enhancement was found: in three-year follow-up the difference in re-employment between the intervention group and the control group was insignificant. When participants with optimal and suboptimal baseline health were analysed separately, re-employment tended to be more prevalent among participants with optimal health in the intervention group, whereas among the participants with suboptimal health re-employment tended to be lower in the intervention group. However, these differences were not statistically significant.

Different interpretations may be made on the result, which tended to contradict rather than support, the expected improvements in the re-employment. How is it possible that the intervention seemed to harm rather than to benefit those in particular need of health care, i.e. those with poor health? One explanation may be in the health related selection at recruitment. Indicative of this, self-rated health was more commonly poor in the intervention group. Moreover, as participation was voluntary, the groups may have differed with respect to their motives to participate. In other words, in the intervention group the participants with suboptimal health may have had relatively severe health problems as regards employability. An alternative explanation is that this kind of health care is not able to improve health and consequent re-employment. As we have reported elsewhere [18, 26] several logics are present in CHC, and this is why the service model adopted from occupational health care does not seem to work in the context of unemployment. Moreover, it is possible that the intervention maintained and strengthened elements of the sick role, instead of enhancing the role as a capable job seeker.

OHC is obliged to support employees with impaired work ability to cope at the workplace, in other words, to prevent health related selection out of work. Analogously, CHC should succeed in promoting re-employment in particular among the unemployed with impaired employability. In this respect, the result of our trial was discouraging. We would like to agree with the view [27] that, even when tailored to meet the assumed needs of a specific client group, the health service mobilizes them only partly, and not all of them benefit from it. To provide a service that matches the increasing diversity of contemporary labour market trajectories is complex. In addition to mere ‘guaranteed access’ to existing or novel service systems, attention should be paid explicitly to their orientation and contents. Our intervention was carried out in the frames of an established OHC service. If the results had been more promising, it would have been possible to conclude that it is not too problematic to transfer and adjust the model for participants in ALMP measures.

The design with a long follow-up time, large intervention and control groups with reasonable sample attrition may be considered to be among the strengths of this study. The established health indicators also reflected different aspects of mental well-being. Although stratification of the participants by baseline health did not reveal statistically significant differences, it deepened the analysis into potential effects that would have otherwise gone unnoticed. A general (level) limitation is that such intervention studies tend to be inherently national and particular with respect to the participant groups and details of the intervention. With respect to earlier research [14, 28], the target group of this study represented less severely marginalised group of the unemployed. Moreover, participation in the study was voluntary, e.g. not related with unemployment benefits.

It is evident that existing, more or less universal service systems do not guarantee adequate health promotion for the unemployed. Therefore, ‘positive discrimination’ of the unemployed with regard to improving their health, and consequent re-employment, will continue to be central to the public health and policy agenda in future years. The present study provides research evidence for the choice of policy instrument. Both health services and employment policy services are important for the well-being of the disadvantaged, but they are probably not interchangeable.

Parallel results from Finland and the Netherlands [15] about the ineffectiveness of health services for the unemployed do not justify the conclusion that any service would be ineffective. Rather, there is a need to develop innovative service contents and models in this field in different national contexts. Alternatively, any particular service tends to stigmatize the client group, and an appropriate direction might rather be to concentrate on developing universal health care and the skills of all professionals with respect to unemployed people.

## Conclusions

The experimental health service did not show any beneficial effects on re-employment. Nevertheless, rather than considering any particular health care as unnecessary and ineffective, we would like to stress the complexity of providing health services to match the diversity of the unemployed.
